# Evolution Toward Severe Covid-19 From Biological Monitoring to Therapeutic Considerations

**DOI:** 10.3389/fimmu.2020.562038

**Published:** 2020-12-15

**Authors:** Julien Carvelli, Audrey Le Saux, Jeremy Bourenne, Marc Gainnier, Gilles Kaplanski

**Affiliations:** ^1^Réanimation des Urgences, CHU Timone, AP-HM, Aix-Marseille Université, Marseille, France; ^2^Marseille Immunopôle, CHU Timone, AP-HM, Marseille, France; ^3^Médecine Interne et Immunologie clinique, CHU Conception, AP-HM, Aix-Marseille Université, Marseille, France

**Keywords:** COVID-19, SARS-CoV-2, C reactive protein, IL-6, cytokine storm

## Introduction

Siddiqi and Mehra (1) recently proposed three stages of COVID-19 with related therapeutic strategies. Stage I (mild) is the early phase of the disease, corresponding to primary invasion by SARS-CoV-2, generally with few symptoms and a spontaneous favorable outcome for almost 85% of patients. Fifteen percent get worse. These latter patients progress to stage II (moderate pulmonary involvement) or III (severe systemic hyperinflammation). Pneumonia, acute respiratory distress syndrome (ARDS), and possible associated organ failures result in their admission to the intensive care unit (ICU). Stage II and III are related to a pathological host-immune response, characterized by a cytokine storm. The so-called cytokine storm is associated with the most severe forms of COVID-19 (2). The IL-6 pathway likely plays a central role in such pathogenesis (3), as reflected by the level of *C-Reactive Protein* (CRP), which is a marker of IL-6 activation (4).

In our ICU, we managed 18 stage II or III patients with severe COVID-19. We screened naso-pharyngeal samples by SARS-CoV-2 rt-PCR at least once a week (88 values) and measured their corresponding plasma levels of CRP (101 values). *Ethics*: observational study, CPP Ile-de-France III 26/03/3020 (2020-A00757-32).

**Figure 1:**
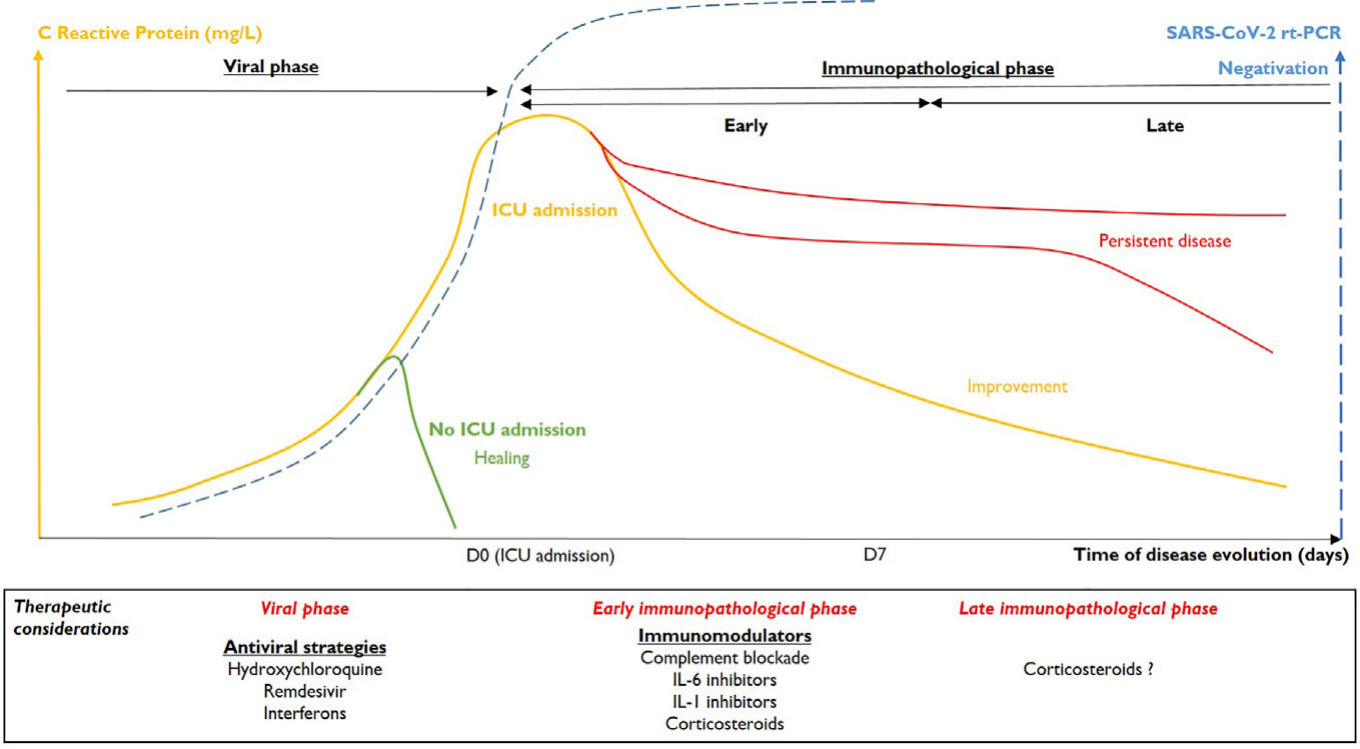
Kinetics of severe Covid-19 and related therapeutic considerations.

The concomitant evolution of the rt-PCR and CRP results were analyzed. Based on these data, we defined two phases of severe COVID-19 (**Figure 1**).

## First Phase: The Viral Phase

COVID-19 begins by the infection of the human airway epithelium through ACE2 receptors (5). Stage I is characterized by non-specific symptoms, such as fever, cough, flu-like syndrome, or anosmia, etc. and virus is detectable by rt-PCR in naso-pharyngeal samples. Antiviral therapies could be useful at this stage, even if 85% of patients recover spontaneously (proportionate and effective immune response) and no antiviral drug has yet proven efficacy (6, 7). Fifteen percent of patients worsen and develop pneumonia or another organ dysfunction (stage II) that could lead them to ICU.

## Second Phase: The Immunopathological Phase

The second step is characterized by organ dysfunction with endothelialitis (8) and is mainly due to an excessive and persistent immune response to infection (2), rather than to viral replication. As mentioned above, these patients may in large parts be distinguished through their CRP concentrations. Inflammation mainly affects the lungs (pneumonia, ARDS), but is likely to be systemic, simultaneously affecting other organs (acute kidney injury, myocarditis, shock, etc.). We believe that two dynamic phases can be distinguished in this stage.

### Early Immunopathological Phase

The transition from viral phase to early immunopathological phase is characterized by progression of the inflammatory process, shown by increased CRP levels. The peak CRP value for our patients correlated with maximum organ injuries (CRP/SOFA correlation). Three levels of severity could be defined: pneumonia without ARDS requiring low oxygen (<5 L/min), ARDS justifying increasing oxygen requirements or even mechanical ventilation and finally systemic involvement with at least one organ failure associated with acute lung injury (stage III of Siddiqi and Mehra). Increased CRP levels are accompanied, in most of the cases, by the negativation of SARS-CoV-2 rt-PCR in pharyngeal samples. In our ICU, COVID-19 patients with negative pharyngeal SARS-CoV-2 rt-PCR are frequently observed and do not correspond to false-negative patients but to patients getting in early immunopathological phase.

Immunomodulators should be proposed at this stage. The anti-IL-6 receptor mAb, *tocilizumab*, could be an option (9). However, *tocilizumab* is difficult to handle in the ICU because of its long half-life (more than 6 days). Physicians could also consider the IL-1Receptor antagonist, *anakinra* (10). *Anakinra* is a powerful inhibitor of both IL-1a and IL-1b, and IL-1b processed through the NLRP3 inflammasome activation has been shown to be involved in coronaviruses-related lung injuries (11). IL-18 is also processed by the NLRP3 inflammasome and activation of the IL-1/IL-18 pathway (2, 12) in our patients, is suggested since they demonstrated high serum ferritin levels. The complement system is also involved in the pathophysiology of severe COVID-19 (8, 13) and complement inhibitors, such as avdoralimab (anti-C5aR1 mAb) are also in evaluation. Downregulation of kinin signaling pathway and contact phase coagulation could also prevent capillary leak-related ARDS, and microthrombi (14, 15). All these strategies must be validated by prospective randomized clinical trials and so far, only the non-specific dexamethasone has shown little efficacy at this stage of the disease (16). Immunomodulator efficacy on severe COVID-19 will be arduous to prove due to their immunosuppressive effects, leading to increased morbidity, such as observed in septic patients.

### Late Immunopathological Phase

In the latter patients, the cytokine storm may persist. High levels of CRP > 7 days could indicate an unfavorable outcome, with the persistence of pulmonary failure (corresponding to fibroproliferation or cystic fibrosis) and multiorgan failure. Among our patients, three had an unfavorable respiratory outcome (MV > 28 days), with signs of fibroproliferation or pulmonary cystic necrosis on CT-scans. In the majority of cases, CRP levels in these patients were very high (> 100–200 mg/L) for more than one week. No uncontrolled bacterial sepsis was identified at the time of CRP sampling in our patients. Anti-cytokine treatments or corticosteroids could constitute a rescue therapy at this stage (pulmonary fibrosis). However, immunotherapies that dampen inflammation should be administered earlier to prevent such an evolution. Better evolution in immunopathological phase is indicated by a more rapid decrease in inflammation, with a rapid decrease of CRP levels.

## Discussion

Our proposal is to distinguish two phases in the kinetics of severe COVID-19 (17). The viral phase may respond to antiviral therapies. The immunopathological phase begins with organ failure, dominated by acute pulmonary failure. The therapeutic objective in ICU, using immunotherapies, would be to control the inflammatory response in order to reduce the evolution toward multiple organ failure and pulmonary fibrosis, without favoring viral recurrence. In the absence of clear prognostic parameters, immunotherapies should most probably be initiated early in the immunopathological phase to be effective and well tolerated.

## Author Contributions

JC devised and supervised the study, designed the research, and wrote the manuscript, with the help of the other co-authors AS, JB, MG, and GK. GK provided key expertise. All authors contributed to the article and approved the submitted version.

## Conflict of Interest

The authors declare that the research was conducted in the absence of any commercial or financial relationships that could be construed as a potential conflict of interest.
