# Supplementary figures and images for: Evolution Toward Severe Covid-19 From Biological Monitoring to Therapeutic Considerations
Source: Front Immunol. 2020 Dec 15;11:562038. doi: 10.3389/fimmu.2020.562038 (PMC7770161; doi:10.3389/fimmu.2020.562038)

## Slide 1
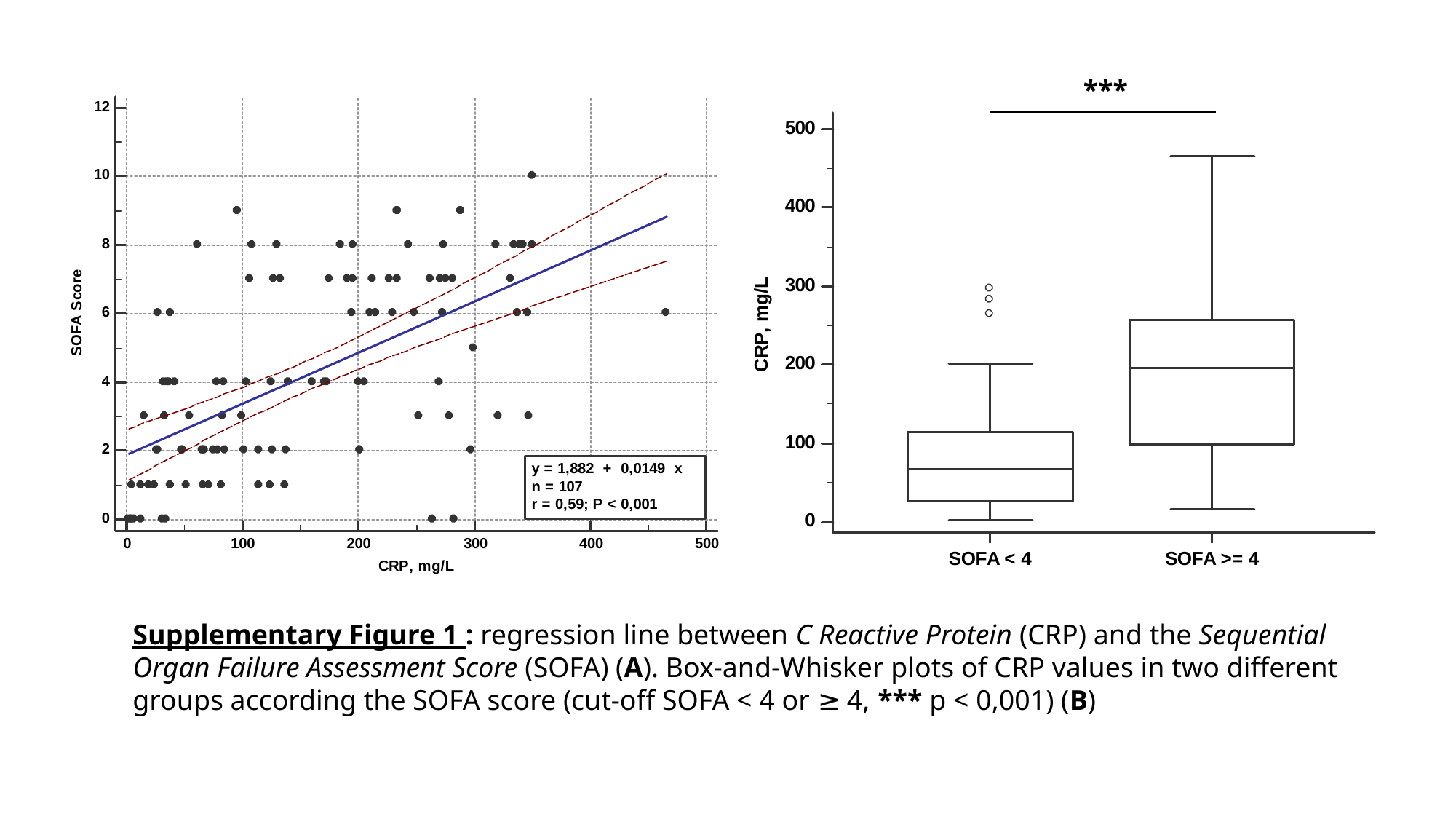

## Slide 2
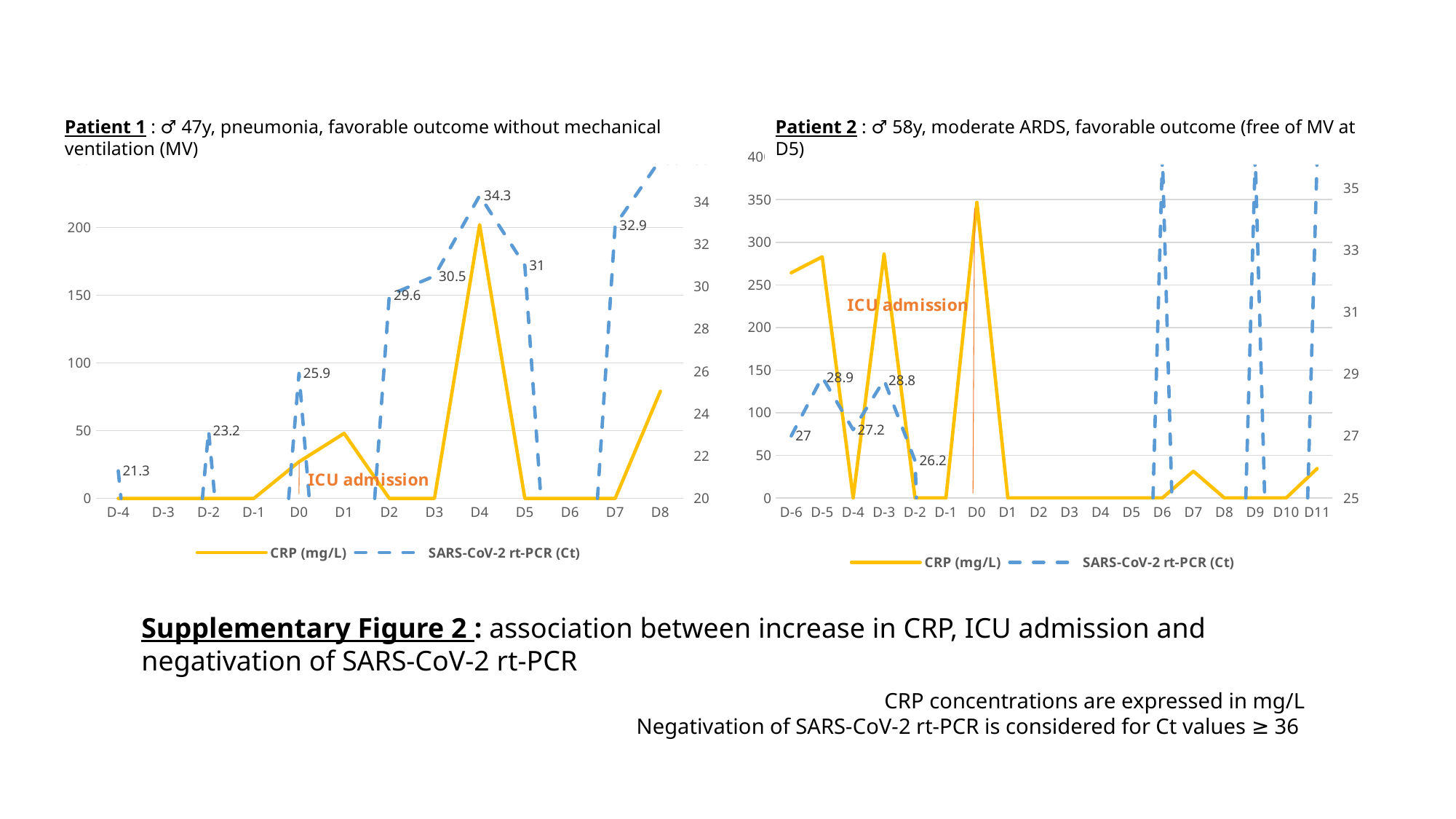

## Slide 3
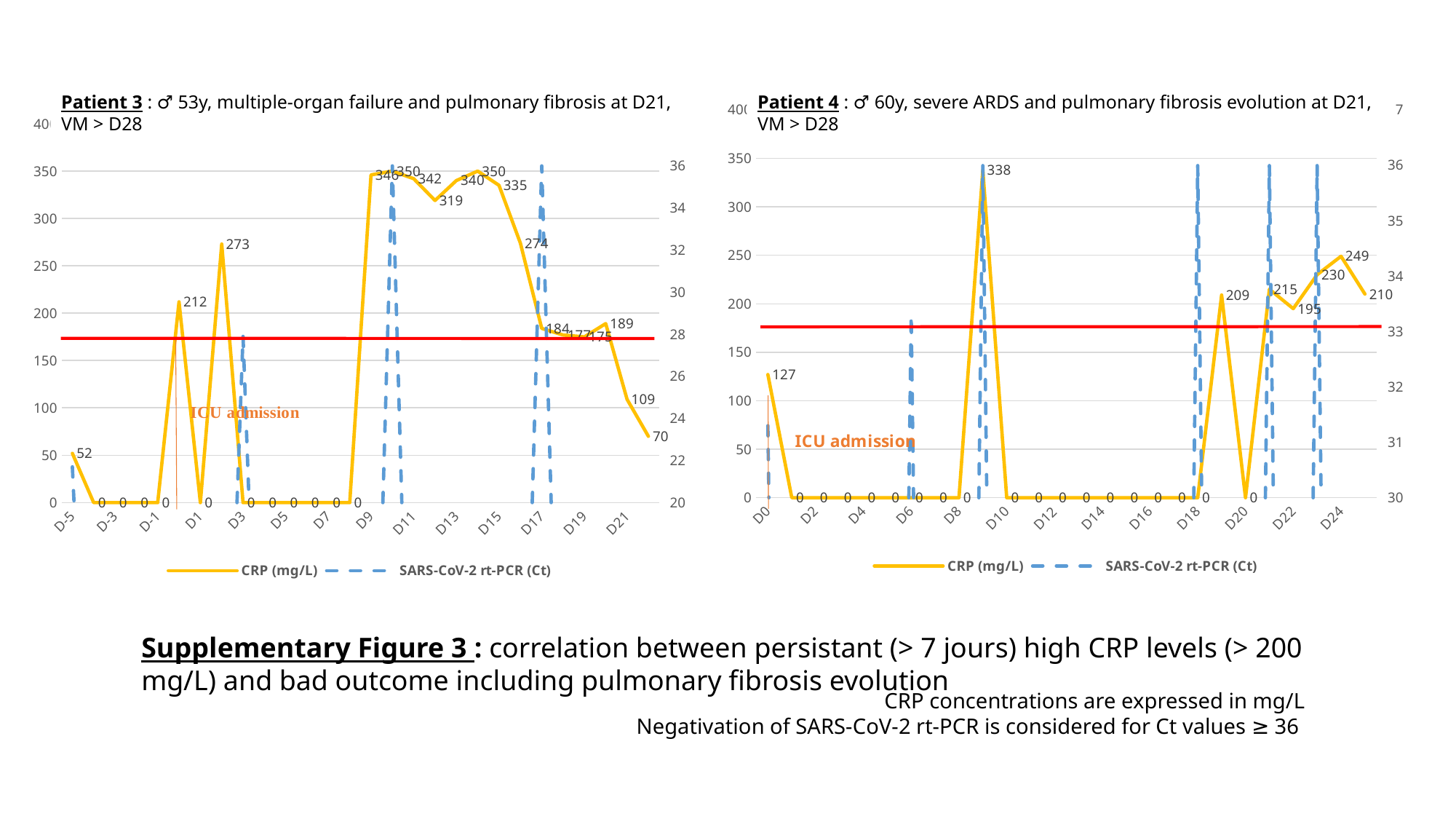

Supplement: Supplementary file 1 [file Presentation_1.pptx]
